# *N*-Methyl- and *N*-Phenylpiperazine Functionalized Styryl Dyes Inside Cucurbiturils: Theoretical Assessment of the Factors Governing the Host–Guest Recognition

**DOI:** 10.3390/molecules28248130

**Published:** 2023-12-16

**Authors:** Nikoleta Kircheva, Vladislava Petkova, Stefan Dobrev, Valya Nikolova, Silvia Angelova, Todor Dudev

**Affiliations:** 1 Institute of Optical Materials and Technologies “Acad. J. Malinowski”, Bulgarian Academy of Sciences, 1113 Sofia, Bulgaria; nkircheva@iomt.bas.bg (N.K.); vpetkova@iomt.bas.bg (V.P.); sdobrev@iomt.bas.bg (S.D.); sea@iomt.bas.bg (S.A.); 2 Faculty of Chemistry and Pharmacy, Sofia University “St. Kliment Ohridski”, 1164 Sofia, Bulgaria; ohtvd@chem.uni-sofia.bg; 3 University of Chemical Technology and Metallurgy, 8 St. Kliment Ohridski Blvd, 1756 Sofia, Bulgaria

**Keywords:** *N*-methylpiperazine, *N*-phenylpiperazine, styryl dye, cucurbit[n]uril, host–guest complex, host–guest recognition

## Abstract

The family of cucurbiturils (CBs), the unique pumpkin-shaped macrocycles, has received great attention over the past four decades owing to their remarkable recognition properties. They have found diverse applications including biosensing and drug delivery technologies. The cucurbituril complexation of guest molecules can modulate their pK_a_s, improve their solubility in aqueous solution, and reduce the adverse effects of the drugs, as well as enhance the stability and/or enable targeted delivery of the drug molecule. Employing twelve cationic styryl dyes with N-methyl- and N-phenylpiperazine functionality as probes, we attempted to understand the factors that govern the host–guest complexation of such molecules within CB[7] and CB[8] host systems. Various key factors determining the process were recognized, such as the pH and dielectric constant of the medium, the cavity size of the host, the chemical characteristics of the substituents in the guest entity, and the presence/absence of metal cations. The presented results add to our understanding (at the molecular level) of the mechanism of encapsulation of styryl dyes by cucurbiturils, thus shedding new light on various aspects of the intriguing complexation chemistry and the underlying recognition processes.

## 1. Introduction

Ribonucleic acid (RNA) and deoxyribonucleic acid (DNA) attract immense interest from scientists of diverse fields not only because they carry the fundamental genetic information in almost all living organisms, but also due to their irreplaceable functions in catalysis, intracellular recognition, and transport [1]. The so-called quadruplexes: noncanonic DNA/RNA motifs of recent finding part of the telomeres have further been recognized as plausible drug targets especially in antitumor therapy [2,3,4]. Their complex architecture is formed by folding guanine-rich nucleic acid sequences into square–planar compositions arranged into stacked layers (tetrads) additionally stabilized by the presence of alkali metal cations, most commonly potassium or, less frequently, sodium located in the central channel [5,6,7,8]. Selectively binding small heterocyclic molecules to G-quadruplexes could decrease telomerase activity and increase telomere stability, reduce oncogene expression by inhibiting transcription and translation, and increase genome instability, hence, prompting apoptosis (cell death) [9,10,11]. In this regard, cyanine dyes have been acknowledged for their beneficial properties, as they exert a dual effect upon polynucleotide binding/cell entering: by triggering specific spectroscopic response, they act simultaneously as imaging and therapeutic agents [12,13]. Representatives of the subgroup called hemicyanines—the cationic form of styryl dyes (incorporating the univalent C_6_H_5_-CH=CH- styryl moiety)—should further be outlined as promising theranostic reagents [14]. Depending on their substituents, certain styryl dyes resemble the classical cyanine molecules in that they possess two nitrogen atoms connected through a conjugated chain of double bonds, but differ in that one of the N-atoms is not part of the heterocyclic nucleus. Most frequently, styryl dyes exist in their cationic form due to the presence of a quaternized nitrogen in the heterocycle. Their current application in dying different nucleic acids by intercalating or groove binding comes as the result of the systematic work by Yarmoluk et al. [15,16], who provided strong evidence of their increased fluorescence in the presence of DNA. Further investigation, however, is required in order to modify their unwanted characteristics and additionally amplify the positive qualities of this class of molecules.

Encapsulating compounds of major significance (“guests”) by specific molecules (“hosts”) encompasses a relatively new and fast developing branch of chemistry known as “supramolecular chemistry”. Geometrical and chemical compatibility of the building fragments allows them to assemble into more complex structures, thus broadening the field of application of both participants in pharmacy (targeted drug design, decreasing adverse effects), food industry (masking unpleasant taste and/or smell), catalysis (enzymatic assays), photochemistry (on/off switches), etc. [17,18,19]. Among many host molecules, e.g., cyclodextrins [20,21,22,23], calixarenes [24,25], crown ethers [26,27], the cucurbituril (CB) family falls into the scope of the present study due to their appealing properties and ever-growing application [28,29,30,31,32,33]. CBs are produced through the condensation of glycoluril with formaldehyde in a relatively simple chemical reaction. The number of involved glycoluril units, *n*, from 5 to 10 (without 9) and 13 to 15, defines the widely accepted labeling of the macrocycle as CB[*n*], which further corresponds to the width of the CB ring [34,35,36]. Interestingly, all cucurbiturils are rigid highly symmetrical structures that share the same height of 9.1 Å, but differ in the cavity diameter (and hence in cavity volume). They possess hydrophobic inner space with low polarity and polarizability; however, their carbonyl-laced portals attract positively or partially positively charged molecules through ion–dipole or dipole–dipole interactions. Although CB[6], CB[7], and CB[8] possess cavities of similar size and are therefore analogous to α-, β-, and γ-cyclodextrins, respectively, they bind guest molecules much stronger, exhibiting several orders of magnitude higher binding constants, particularly in the case of positively charged guests [37,38,39].

**Figure 1:**
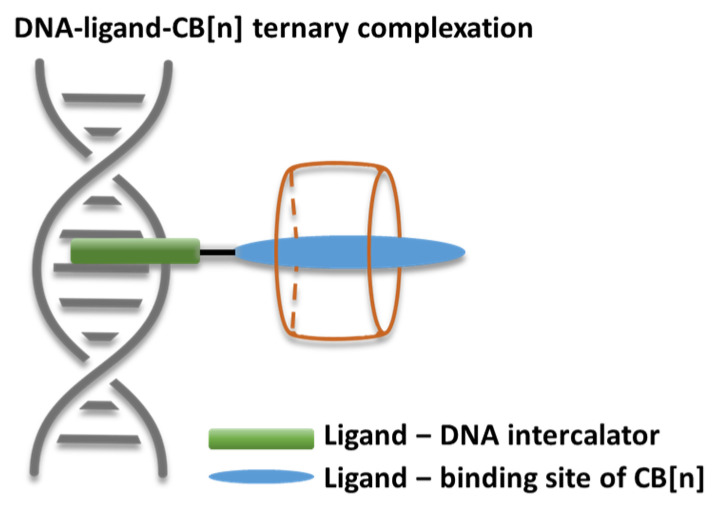
Schematic presentation of a ternary complex between DNA, a small heterocyclic ligand, and a CB[n].

The current research is focused on the encapsulation of a series of styryl dyes previously suggested and investigated by Zonjić et al. [40] in the cavity of CB[7] and CB[8]. As reported in earlier work [41,42,43,44], encasing styryl dyes in cucurbiturils positively affects their action as DNA intercalators and quadruplex stabilizers, as well as enhances the fluorescence yield. A schematic presentation of a ternary complex between DNA, a heterocyclic guest, and a CB[n], illustrating their mode of action, is presented in Figure 1. Hence, it is of great significance to determine the best reaction conditions and important factors that govern the host–guest recognition process. Applying the in silico approach prior to experimental work is therefore beneficial. The presented study aims at disclosing factors of high importance that play a key role in the modeled reactions of encapsulation such as the cavity volume, pH, and dielectric constant of the medium, presence of metal cations in the reaction medium, and specific chemical characteristics of different substituents in the dye molecule. The previously implemented well-tested DFT procedure [45,46,47,48,49] provides clear trends and reliable results in agreement with published experimental data.

## 2. Results and Discussion

### 2.1. Reactions Modeled

**Figure 2:**
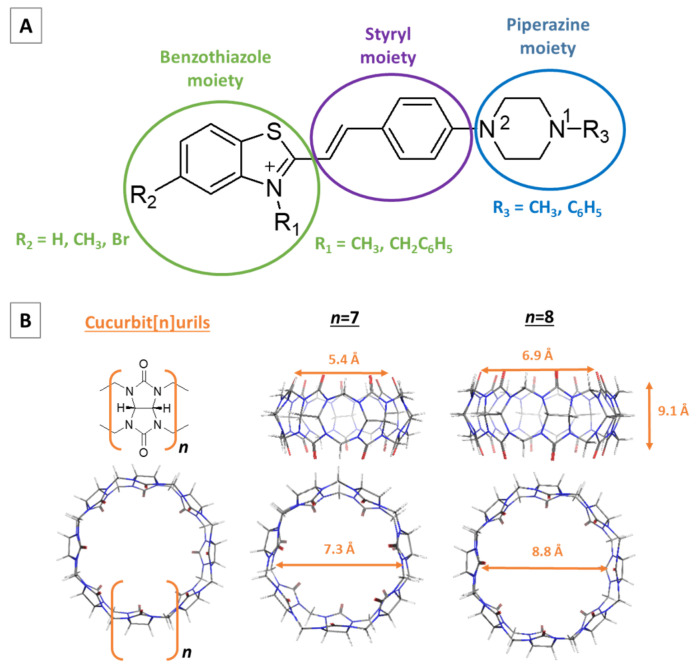
Generalized structure of the styryl-based dyes under study. Each structure is further presented in Table 1 according to the type of R_1_-/R_2_-/R_3_- substituents (**A**). Chemical structure of the glycoluril unit, CB[7] and CB[8] (**B**). The dimensions of CB[7] and CB[7] are reported in [29,31].

The current study is aimed at providing reliable trends concerning the host–guest recognition process between *N*-methyl- and *N*-phenylpiperazine functionalized styryl dyes in their monocationic form and cucurbit[7/8]uril, further denoted as CB[7] and CB[8]. In order to accomplish the task, the structures presented in Figure 2A and Table 1, first suggested by Zonjić and co-authors in Ref. [40], were considered. Three moieties build the dye molecules: a styryl moiety, which is conserved in all structures; a benzothiazole moiety, containing R_1_-(being either a hydrogen, a methyl group, or a bromine atom) and R_2_-substituents (a methyl or a benzyl group), and a piperazine moiety, differing in the nature of the R_3_-substituent being either a methyl- or phenyl group. Thus, the possible combinations result in overall 12 dyes, henceforth referred to as the more generalized dyeN nomenclature, where N stands for the numbers from 1 to 12, e.g., Dye1–12. The *N*-methylpiperazine functionalized structures correspond to the numbers from 1 to 6, while to their equivalent *N*-phenylpiperazine dyes have been assigned the numbers from 7 to 12. The chemical structures of the host molecules, their spatial parameters (height, diameter of the inner cavity, and distance between the carbonyl groups of the outer rim), and the glycoluril unit are additionally presented in Figure 2B.

For the assessment of the factors governing the host–guest recognition, the following reactions were taken into account:

CB[7/8] + dyeN^+^ → CB[7/8]@dyeN^Ⴈ+^ (R1)

CB[7]/CB[7]@8W + dye9^+/2+^ → CB[7]@dye9^Ⴈ+/2+^ + 8W (R2)

CB[7]@dye9^Ⴈ+^ + Mg^2+^→ CB[7]@Mg^Ⴈ2+^ + dye9^+^ (R3)

CB[7]@dye9^Ⴈ+^ + Mg^2+^→ CB[7]@dye9@Mg^Ⴈ3+^ (R4)

Reaction R1 illustrates the encapsulation of all the considered dyes in their monocationic form in both cucurbiturils. This approach allows the disclosure of important factors that affect the process, such as the cavity volume, nature of the R_1_-, R_2_-, and R_3_-substituents, and dielectric constant of the environment. Reactions from R2 to R4 consider the complexation of the most intriguing of the representatives of the series—dye9, in regard to obtained results in Ref. [40] and in the current research, and are focused on the assessment of the effect of pH of the medium, charge of the molecule, presence of high-energy water cluster in the host, and of magnesium cations in the solution. Mg^2+^ stands for both a bare cation and one surrounded by a hydration shell of six water molecules, in which case the ternary complex has the formula CB[7]@ dye9@Mg_2W^Ⴈ3+^ after the loss of four water molecules (denoted as 4W) in reaction R4. Reaction R3 exemplifies the role of the metal cation as a competitor for binding the CB[7] (substitution), while reaction R4 portrays the formation of a ternary complex (addition).

### 2.2. Encapsulation of Dyes1-12^+^ in CB[7/8]

#### 2.2.1. Effect of the Substituents

**Figure 3:**
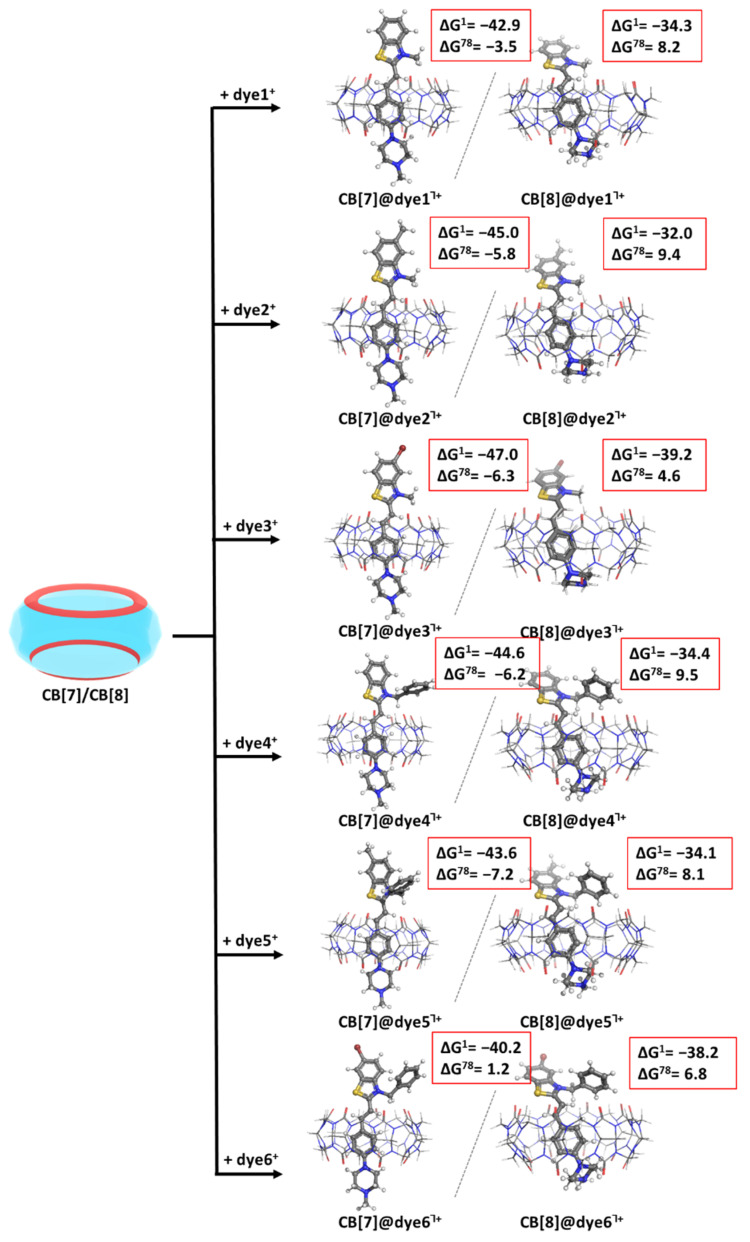
Optimized structures in the gas phase at the M062X/6-31G(d,p) level of theory of the CB[7]@dye1–6^Ⴈ+^ (first column) and CB[8]@dye1–6^Ⴈ+^ (second column) complexes, along with the corresponding ∆G^ε^ values in kcal mol^−1^ for their formation. The upper index indicates a reaction in the gas phase (ε = 1), and in a water environment (ε = 78) yielded at the M062X/6-31+G(d,p)//M062X/6-31G(d,p).

The process of encapsulation of Dyes1-6^+^ in CB[7/8] is depicted in Figure 3 (first/second column, respectively). The optimized structures of the resulting complexes at the M062X/6-31G(d,p) along with the Gibbs energies (in kcal mol^−1^) of their formation at the higher M062X/6-31+G(d,p)//M062X/6-31G(d,p) level are given as well.

The obtained results imply that the formation of CB[7]@dye1–6^Ⴈ+^ complexes appears thermodynamically favorable for almost all styryl dyes as the Gibbs energies stay firmly on negative ground: the presented data range from −3.5 to −7.2 kcal mol^−1^ (Figure 3, first column). The only exception is the recognition process between CB[7] and dye6^+^, where the calculated ∆G^78^ value equals 1.2 kcal mol^−1^, which still remains close to zero and is within the acceptable error of the computational method. The inclusion process in the more voluminous CB[8], however, is thermodynamically improbable as the obtained ∆G^78^ values are positive and vary between 4.6 and 9.5 kcal mol^−1^ (Figure 3, second column).

**Figure 4:**
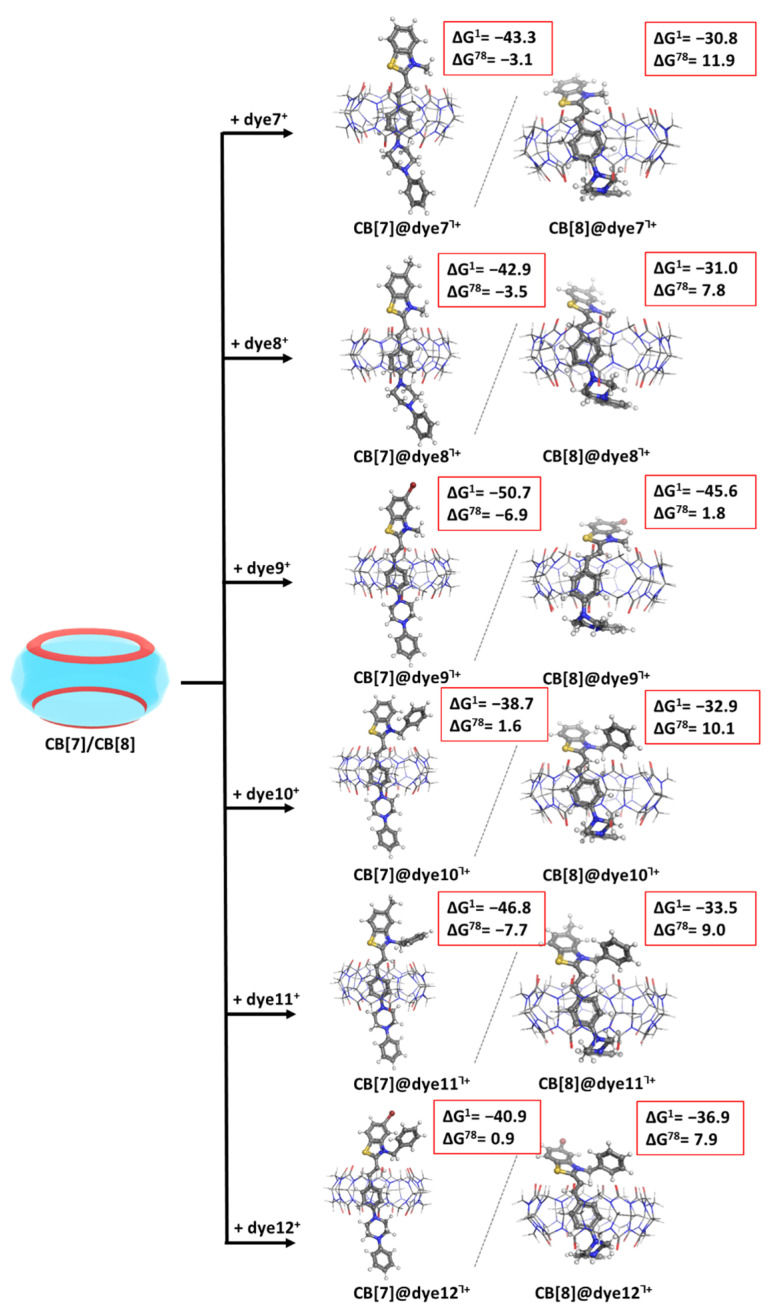
Optimized structures in the gas phase at the M062X/6-31G(d,p) level of theory of the CB[7]@dye7–12^Ⴈ+^ (first column) and CB[8]@dye7–12^Ⴈ+^ (second column) complexes, along with the corresponding ∆G^ε^ values in kcal mol^−1^ for their formation. The upper index indicates a reaction in the gas phase (ε = 1), and in a water environment (ε = 78) yielded at the M062X/6-31+G(d,p)//M062X/6-31G(d,p).

The following Figure 4 depicts the encapsulation process between the remaining dyes7–12^+^ and the two host cucurbiturils.

The calculations indicate a similar trend for the *N*-phenylpiperazine functionalized styryl dyes: the encapsulation in CB[7] is a thermodynamically probable process evidenced by the negative ∆G^ε^ values in almost all cases (except for dye10 and dye12, where the evaluated free energies, although positive, stay close to zero): see the first column in Figure 4. On the other hand, the reactions of formation of CB[8]@dye7–12^Ⴈ+^ do not occur spontaneously under the simulated conditions, as all of the ∆G^ε^ values vary between 1.8 and 11.9 kcal mol^−1^: see the second column in Figure 4.

The presented results draw some clear trends in regard to the nature of the substituents in the dyes’ molecules. Firstly, the methyl/phenyl group at R_3_ position does not substantially affect the outcome of the host–guest recognition in CB[7], as the obtained ∆G^ε^ values fall in close ranges: from 1.2 to −7.2 kcal mol^−1^ for the formation of complexes CB[7]@dye1–6^Ⴈ+^, and from 1.6 to −7.7 kcal mol^−1^ for the formation of the analogous complexes CB[7]@dye7–12^Ⴈ+^, with a minor preference of up to 0.6 kcal mol^−1^ for the smaller group. The only odd exception strikes the difference in the recognition between CB[7] and dye4/dye10. These two structures are equivalent in regard to R_1_ and R_2_ but differ by 7.8 kcal mol^−1^ in their ∆G^ε^ values. This result can be explained by the position of the dyes in the host cavity: while the N_2_-atom from the piperazine moiety in dye4 is incorporated in relatively close proximity to two carbonyl groups at 3.4/3.2 Å, the equivalent N_2_-atom in dye10 interacts with only one C=O group located at 3.3 Å. This composition results in the loss of dipole–dipole interaction and, hence, unfavorably affects the encapsulation of dye10 in CB[7] (positive ∆G^78^ = 1.6 kcal mol^−1^). Furthermore, the R_1_ substituent more strongly influences the complexation between CB[7] and all the dyes, as expected, since it is the group directly bound to the quaternized N-atom from the benzothiazole moiety. The benzyl group exerts a stronger beneficial impact upon the host–guest recognition, as indicated by the higher absolute values of the calculated ∆G^ε^. The explanation lies in the greater negative inductive effect of the benzyl moiety as compared to its methyl counterpart, resulting in drawn electronic density from the N^+^ and enhanced ion–dipole interaction with the carbonyl groups from the host. Additionally, the CH_3_-group being smaller manages to insert between the N^+^ ion and the C=O groups from the cucurbituril rim, consequently enhancing the distance between them. Lastly, the R_2_ substituents affect the complexation in CB[7] in a clear way: with the substitution of H→CH_3_→Br, the obtained ∆G^ε^ values increase in absolute value (the recognition process becomes more favorable). The only combination that appears disadvantageous is between the benzyl-moiety and the bromine atom: positive Gibbs energies for the formation of CB[7]@dye6/12^Ⴈ+^ complexes due to the opposite directions of their effects.

Similar (but not necessarily in all cases) trends are observed for the encapsulation of the dyes in CB[8] with regard to the effect of the substituents. Among the R_1_ substituents, the direction of the most beneficial is H < CH_3_ < Br. The methyl group at R_2_ position leads to better results as compared to the benzyl substituent. Overall, the *N*-methyl functionalized styryl dyes are less affected by the change of the substituents at the other positions since the obtained results fluctuate less in comparison with the calculated ∆G^ε^ values for the formation of CB[8]@dye7–12^Ⴈ+^: from 4.6 to 9.5 kcal mol^−1^ vs. from 1.8 to 11.9 kcal mol^−1^, respectively. Yet, all the obtained Gibbs energies stand strongly on positive ground, indicating a thermodynamically improbable reaction.

#### 2.2.2. Effect of the Cavity Volume

The effect of the cavity volume is unambiguous—the *N*-methyl- and *N*-phenylpiperazine functionalized styryl dyes under study prefer the smaller cavitand, the preference being indicated by the negative ∆G^ε^ values in almost all cases for their encapsulation in CB[7] as opposed to the positive Gibbs energies obtained for the complexation in CB[8]. These results should be attributed to the lost interaction between the host and the guest molecules in the case of the more voluminous representative. Note that this outcome falls in line with experimental observations that CB[7] incorporates dyes in a 1:1 ratio, while CB[8] readily forms complexes but with two dye molecules in the cavity corresponding to a 2:1 or even 2:2 ratio [50,51,52].

#### 2.2.3. Effect of the Dielectric Constant of the Medium

The presented results are given for two media—gas phase (close to a nonpolar solvent/protein environment) and water. By the implementation of the thermodynamic cycle for calculating the contribution of the solvation energy, the effect of the dielectric constant of the medium can be assessed. The balance between energy gain and de/solvation effects defines the outcome of the complexation process, as seen Figure 3 and Figure 4. For instance, the recognition between CB[8] and the modeled dyes appears possible in the gas phase (negative ∆G^1^ values); this trend changes when the reaction takes place in aqueous solution.

### 2.3. Encapsulation of Dye9 ^Ⴈ+/2+^ in CB[7] in Absence/Presence of Metal Cations

#### 2.3.1. Effect of the pH of the Medium/Charge of the Dye Molecule

**Figure 5:**
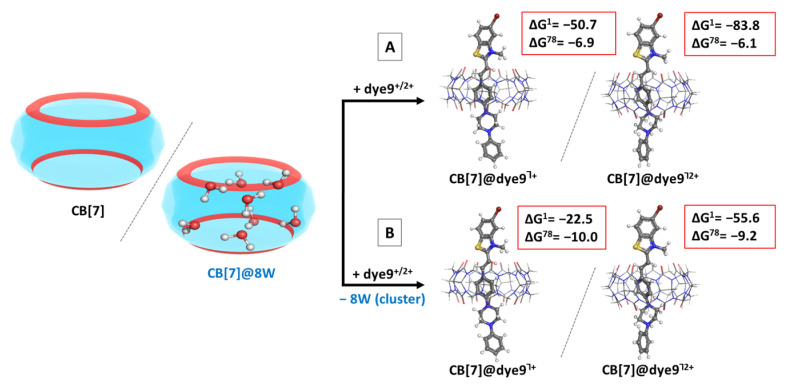
Optimized structures in the gas phase at the M062X/6-31G(d,p) level of theory of the CB[7]@dye^Ⴈ+^ (first column) and CB[7]@dye9^Ⴈ2+^ (second column) complexes, along with the corresponding ∆G^ε^ values in kcal mol^−1^ for their formation. The results are obtained by modeling either an “empty” host (**A**) or a cucurbituril with high-energy water molecules (**B**) as an initial structure. The upper index indicates a reaction in the gas phase (ε = 1) and in a water environment (ε = 78) yielded at the M062X/6-31+G(d,p)//M062X/6-31G(d,p) level.

For the assessment of factors such as the pH of the medium/charge of the dye molecule, reaction R2 was simulated. The optimized structures of dye9 in its mono- (denoted as dye9^+^) and dicationic (denoted as dye9^2+^) forms participate in the reaction and their structures are explicitly presented in Figure S1. The obtained data-optimized binary host–guest complexes and ∆G^ε^ values in different media are presented in Figure 5A—with an initial “empty” host, and in Figure 5B—when a high-energy water cluster is present in the cavity.

The calculations indicate that the lower pH of the medium corresponding to a higher charge of the dye does not affect the host–guest recognition process significantly as the results differ by 0.8 kcal mol^−1^: compare the ∆G^ε^ values for the formation of CB[7]@dye9^Ⴈ+/2+^. This outcome seems somehow peculiar at first sight, because it is expected that the guest with the higher charge would better interact with the host due to the stronger ion–dipole interaction. However, the N_1_-atom, the bearer of the second positive charge, is located further away from the carbonyl rim of the cucurbituril (at about 3.7 Å) due to the quaternized N-atom from the benzothiazole ring acting as an “anchor” and predisposing the position of the piperazine moiety. Note that the structure with the second charge occupying N_1_ is, by 12.9 kcal mol^−1^, more stable than its N_2_ counterpart. Additionally, the solvation penalty for the doubly charged construct is greater than for the monocationic composition, evidenced by the significant difference between the ∆G^ε^ values in the gas phase and in aqueous solution: about 32–34 kcal mol^−1^ more for the formation of CB[7]@dye9^Ⴈ2+^ as compared to CB[7]@dye9^Ⴈ+^ (compare the differences in ∆G^1^/∆G^78^ for the modeled reaction in Figure 5, [A]/[B]: 43.8/12.5 (monocation) vs. 77.7/46.4 (dication) kcal mol^−1^). Overall, the results still suggest that the formation of both complexes CB[7]@dye9^Ⴈ+/2+^ is a thermodynamically possible reaction, as the ∆G^ε^ values stay below zero: −6.9/−6.1 kcal mol^−1^, respectively. Taking into consideration a high-energy water cluster present in the inner cavity of CB[7] [53] constructed by 8 H_2_O molecules, as previously reported in Ref. [35], adds about 3.1 kcal mol^−1^ to the energy gained: the calculated ∆G^ε^ values for the formation of both complexes CB[7]@ dye9^Ⴈ+/2+^ become −10.0/−9.2 kcal mol^−1^ (See Figure 5B). This result is consistent with earlier observations [46,47], suggesting that the hydration of the host is beneficial for the incorporation of metal ions and/or dye guests.

#### 2.3.2. Effect of the Metal Cations

**Figure 6:**
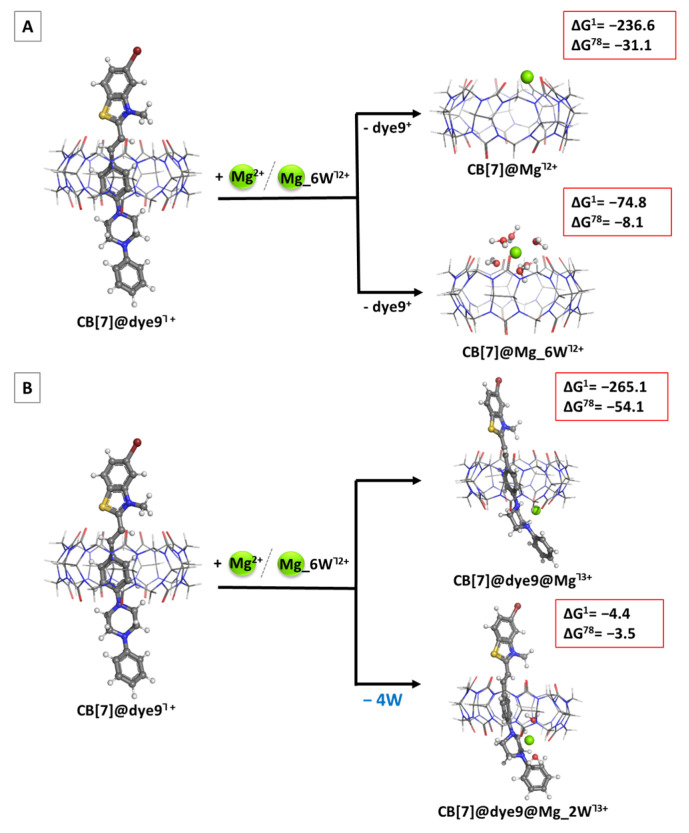
Optimized structures in the gas phase at the M062X/6-31G(d,p) level of theory of the CB[7]@Mg/Mg_6W^Ⴈ2+^ structures in accordance with Reaction 3 (competitive substitution) (**A**) and the ternary CB[7]@dye9@Mg/Mg_2W^Ⴈ3+^ complexes in accordance with Reaction 4 (cooperative addition) (**B**), along with the corresponding ∆G^ε^ values in kcal mol^−1^ for their formation. The upper index indicates a reaction in the gas phase (ε = 1), and in a water environment (ε = 78) obtained at the M062X/6-31+G(d,p)//M062X/6-31G(d,p) level.

The effect of metal cations present in the solution is a subject of great significance for the supramolecular chemistry of cucurbiturils. Since almost all representatives of this class of cavitands are poorly solvated in an aqueous environment, the discovery that alkali metal cations promote the process [54] was an outstanding success for their use in water. It additionally expands their field of application through the formation of novel constructs such as molecular capsules, tubular polymers and molecular jewelry (bracelets and necklaces) [55,56,57,58,59,60]. The metal cation acts as a “lid” that un/blocks the carbonyl portal or can assist with the encapsulation and/or release of the guest molecule under specific conditions, e.g., temperature and/or pH of the medium. Thus, delineating factors of high importance that govern the inclusion of styryl dyes in the CB hosts in the presence of metal cations (in the particular case of Mg^2+^) is mandatory. Based on our previous calculations [61,62], the magnesium cations participating in reactions R3 and R4 were modeled with either a bare or hydrated cations with a shell of six water molecules. Two modes of magnesium interactions were considered: competitive substitution, where Mg^2+^ displaces the bound dye molecule (Figure 6A), and a cooperative addition, where Mg^2+^ co-binds to the already formed cucurbituril–dye complex (Figure 6B).

The obtained results draw a clear picture of the effect of the magnesium cation: it appears beneficial in all the modeled reactions (negative ∆G^ε^ values). Interestingly, comparing the outcome for the bare Mg^2+^, the cooperative addition appears more probable since the corresponding ∆G^78^ are greater in absolute value (−54.1 vs. −31.1 kcal mol^−1^). However, a model closer to reality is the hydrated magnesium cation surrounded by six water molecules. In this case, the Gibbs energies differ by 4.6 kcal mol^−1^ in favor of the competitive substitution. Noteworthy, this conclusion falls in line with other experimental data with different dyes, showing that metal cations more often compete with the guest for binding the cucurbituril in 1:1 ratio, while they assist in the recognition process if two CB[7] molecules incorporate one dye [51].

## 3. Methods

The calculations were performed through the utilization of the Gaussian 09 suite of programs [63]. The most appropriate combination of a method and basis set was found to be the Minnesota functional M062X [64] with the double zeta 6-31G(d,p) basis set applied, and proven to be reliable in our previous investigations of the physicochemical properties of cucurbiturils [45,46,47,49,65]. Initial geometries of the host molecules were derived from TUHGAG (CB[7]) and BATWEA (CB[8]) entries deposited in the Cambridge Crystallographic Data Centre (CCSD) [66,67]. The most stable conformations (chair conformation of piperazine moiety and equatorial positioned N_1_/N_2_-substituents) of *trans* isomers of the dyesN were considered. All structures under study were subjected to full optimization and a vibrational frequency analysis at the lower M062X/6-31G(d,p) level of theory. The latter showed no imaginary frequencies for any of the calculations, thus indicating a local minimum of the potential energy surface (PES). These were used in evaluating the thermal energies, including zero point energy, E_th_, and the entropy, S, while the electronic energies, E_el_, were corrected by single point calculations at the higher M062X/6-31+G(d,p)//M062X/6-31G(d,p) level. In order to calculate the Gibbs energies of the modeled Reactions (1) to (4) in the gas phase corresponding to conditions such as room temperature, T= 298.15K, and atmosphere pressure, 1 atm, these data were implemented in the following equation [68]:

ΔG_1_ = ΔE_el_ + ΔE_th_ + ∆nRT − TΔS (1)

where ΔE_el_, ΔE_th_, and ΔS represent the corresponding differences between the products and the reactants in consistency with Reactions (1) to (4). The change in the number of moles, ∆n, during the reaction is also accounted for in ∆G^1^ (∆nRT ≈ P∆V). Additional single point calculations were performed at the M062X/6-31+G(d,p)//M062X/6-31G(d,p) level in a water environment (ε = 78) using the solvation model based on the density (SMD) method [69]. The respective electronic energies in aqueous environment (E_el_^78^) were used to calculate the solvation energies of the structures as the difference between those in water and those in the gas phase. The overall ∆G^78^ of the reactions were finally determined by summing up the Gibbs energy in the gas phase with the Gibbs energies of solvation of the products, and subtracting the corresponding ∆G^78^ of the reactants. This is expressed by the equation

ΔG^78^ = ΔG^1^ + ΔG_solv_^78^ (products) − ΔG_solv_^78^ (reagents) (2)

Note that a positive ΔG^78^ value indicates a thermodynamically improbable reaction, while a negative one suggests that the encapsulation process happens spontaneously under the reaction conditions. As the conducted study aims at providing reliable trends concerning the factors of high importance that govern the envisioned host–guest recognition, the most significant conclusions should be based not so much on the sole numbers but more on the change/difference between the Gibbs energies when the conditions differ (volume of the cucurbituril cavity, nature of the substituents, pH and dielectric constant of the medium, presence of metal cations, and solvation of the host). The molecular graphics images were created by applying the PyMOL molecular graphics system [70].

## 4. Conclusions

The present calculations shed light on the major factors controlling the encapsulation of styryl dyes by cucurbituril hosts: the smaller cavitand readily binds the modeled guest molecules, while the more voluminous CB[8] does not appear suitable for the recognition process. This outcome should be attributed to the lost interaction between the quaternized N-atom from the benzothiazole moiety and the carbonyl groups from the cucurbituril outer rim due to the bigger diameter of the cavity. Additionally, the dye bends during the optimization in the complex with CB[8], which further affects the final result in a negative manner (positive ΔG^78^ values). As expected, the nature of the substituents, especially those bound to the quaternized N-atom, plays a significant role in the encapsulation process: the benzyl group possesses greater negative inductive effect as compared to its methyl counterpart; hence, it draws electronic density from the benzothiazole N^+^ and enhances the ion–dipole interaction with the carbonyl groups from the host. The *N*-methyl and *N*-phenylpiperazine moieties, although important for the general activity of the series, do not considerably influence the complexation, whereas among the R_1_ substituents, the direction of the most beneficial is H < CH_3_ < Br. The acidity of the medium and the related higher charge of the guest positively affect the encapsulation implied by the negative ΔG^78^ values, but to a lesser than the expected extent due to the greater desolvation penalty of the di-, as compared to the mono-,cationic form. Hence, the polarity of the medium appears to be an immensely contributing factor to the encapsulation process along with the presence of the high-energy water cluster in the host cavity, which adds about 3 kcal mol^−1^ to the energy gain. As reported previously, the tendency of metal cations to rather competitively substitute the bound dye from the cucurbituril cavity when the complex is in a ratio of 1:1 is valid for the studied series of *N*-methyl- and *N*-phenylpiperazine functionalized styryl dyes. Noteworthily, the provided results systematically assess, for the first time (to the best of our knowledge), the factors that govern the incorporation of the studied styryl dyes (with promising DNA/G–quadruplex binding and theranostic properties) inside the CB[7/8] cavity. The conclusions drawn remain firmly in line with earlier reported work for other dyes, but contribute to our understanding of the recognition processes taking place at the molecular level. An intriguing future perspective is undoubtedly the interaction of the binary dye–CB[n] complexes with the intricate structure of DNA by applying docking procedures, molecular mechanics, and/or semiempirical calculations.

## Figures and Tables

**Table 1:** Structures of the dyes under study according to the type of R_1_-/R_2_-/R_3_- substituents.

DyeN	R_1_	R_2_	R_3_	
Dye1	CH_3_	H	CH_3_	*N*-methyl- piperazine styryl dyes
Dye2	CH_3_	CH_3_	CH_3_	
Dye3	CH_3_	Br	CH_3_	
Dye4	Bn	H	CH_3_	
Dye5	Bn	CH_3_	CH_3_	
Dye6	Bn	Br	CH_3_	
Dye7	CH_3_	H	Ph	*N*-phenyl- piperazine styryl dyes
Dye8	CH_3_	CH_3_	Ph	
Dye9	CH_3_	Br	Ph	
Dye10	Bn	H	Ph	
Dye11	Bn	CH_3_	Ph	
Dye12	Bn	Br	Ph	

## Data Availability

The data that support the findings of this study are available from the corresponding author upon reasonable request.

## Supplementary Materials

The following supporting information can be downloaded at: https://www.mdpi.com/article/10.3390/molecules28248130/s1, Figure S1: Optimized structures of dye9 in its mono- and dicationic form.

## References

[1] Blackburn G.M., Gait M.J., Loakes D., Williams D.M. (2006). Nucleic Acids in Chemistry and Biology.

[2] Neidle S. (2016). Quadruplex Nucleic Acids as Novel Therapeutic Targets. J. Med. Chem..

[3] Alessandrini I., Recagni M., Zaffaroni N., Folini M. (2021). On the Road to Fight Cancer: The Potential of G-quadruplex Ligands as Novel Therapeutic Agents. Int. J. Mol. Sci..

[4] Kosiol N., Juranek S., Brossart P., Heine A., Paeschke K. (2021). G-Quadruplexes: A Promising Target for Cancer Therapy. Mol. Cancer.

[5] Smargiasso N., Hsia W., Colson P., Baker E.S., Bowers M.T., Pauw E. (2008). De G-Quadruplex DNA Assemblies: Loop Length, Cation Identity, and Multimer Formation. J. Am. Chem. Soc..

[6] Spiegel J., Adhikari S., Balasubramanian S. (2020). The Structure and Function of DNA G-Quadruplexes. Trends Chem..

[7] Neidle S. (2009). The Structures of Quadruplex Nucleic Acids and Their Drug Complexes. Curr. Opin. Struct. Biol..

[8] Huppert J.L. (2008). Four-Stranded Nucleic Acids: Structure, Function and Targeting of G-Quadruplexes. Chem. Soc. Rev..

[9] Luedtke N.W. (2009). Targeting G-Quadruplex DNA with Small Molecules. Chimia.

[10] Kaneti J., Kurteva V., Georgieva M., Krasteva N., Miloshev G., Tabakova N., Petkova Z., Bakalova S.M. (2022). Small Heterocyclic Ligands as Anticancer Agents: QSAR with a Model G-Quadruplex. Molecules.

[11] Frasson I., Pirota V., Richter S.N., Doria F. (2022). Multimeric G-Quadruplexes: A Review on Their Biological Roles and Targeting. Int. J. Biol. Macromol..

[12] Komljenovic D., Wiessler M., Waldeck W., Ehemann V., Pipkorn R., Schrenk H.H., Debus J., Braun K. (2016). NIR-Cyanine Dye Linker: A Promising Candidate for Isochronic Fluorescence Imaging in Molecular Cancer Diagnostics and Therapy Monitoring. Theranostics.

[13] Li Y., Zhou Y., Yue X., Dai Z. (2021). Cyanine Conjugates in Cancer Theranostics. Bioact. Mater..

[14] Deligeorgiev T., Vasilev A., Kaloyanova S., Vaquero J.J. (2010). Styryl Dyes—Synthesis and Applications during the Last 15 Years. Color. Technol..

[15] Volkova K.D., Kovalska V.B., Tatarets A.L., Patsenker L.D., Kryvorotenko D.V., Yarmoluk S.M. (2007). Spectroscopic Study of Squaraines as Protein-Sensitive Fluorescent Dyes. Dye. Pigment..

[16] Balanda A.O., Volkova K.D., Kovalska V.B., Losytskyy M.Y., Tokar V.P., Prokopets V.M., Yarmoluk S.M. (2007). Synthesis and Spectral-Luminescent Studies of Novel 4-Oxo-4,6,7,8-Tetrahydropyrrolo[1,2-a]Thieno[2,3-d]Pyrimidinium Styryls as Fluorescent Dyes for Biomolecules Detection. Dye. Pigment..

[17] Verma R.K., Garg S. (2001). Current Status of Drug Delivery Technologies and Future Directions. Pharm. Technol. On-Line.

[18] Ma X., Zhao Y. (2015). Biomedical Applications of Supramolecular Systems Based on Host–Guest Interactions. Chem. Rev..

[19] Yang H., Yuan B., Zhang X., Scherman O.A. (2014). Supramolecular Chemistry at Interfaces: Host–Guest Interactions for Fabricating Multifunctional Biointerfaces. Acc. Chem. Res..

[20] Wüpper S., Lüersen K., Rimbach G. (2021). Cyclodextrins, Natural Compounds, and Plant Bioactives—A Nutritional Perspective. Biomolecules.

[21] Crini G. (2014). Review: A History of Cyclodextrins. Chem. Rev..

[22] Szejtli J. (1998). Introduction and General Overview of Cyclodextrin Chemistry. Chem. Rev..

[23] Lee J., Lee S.-S., Lee S., Oh H. (2020). Bin Noncovalent Complexes of Cyclodextrin with Small Organic Molecules: Applications and Insights into Host–Guest Interactions in the Gas Phase and Condensed Phase. Molecules.

[24] Homden D.M., Redshaw C. (2008). The Use of Calixarenes in Metal-Based Catalysis. Chem. Rev..

[25] Vicens J., Böhmer V. (1991). Calixarenes: A Versatile Class of Macrocyclic Compounds.

[26] Bukhzam A., Bader N. (2017). Crown Ethers: Their Complexes and Analytical Applications. J. Appl. Chem..

[27] Kralj M., Tušek-Božić L., Frkanec L. (2008). Biomedical Potentials of Crown Ethers: Prospective Antitumor Agents. ChemMedChem.

[28] Barrow S.J., Kasera S., Rowland M.J., Del Barrio J., Scherman O.A. (2015). Cucurbituril-Based Molecular Recognition. Chem. Rev..

[29] Lagona J., Mukhopadhyay P., Chakrabarti S., Isaacs L. (2005). The Cucurbit[n]Uril Family. Angew. Chem. Int. Ed..

[30] Masson E., Ling X., Joseph R., Kyeremeh-Mensah L., Lu X. (2012). Cucurbituril Chemistry: A Tale of Supramolecular Success. RSC Adv..

[31] Lee J.W., Samal S., Selvapalam N., Kim H.J., Kim K. (2003). Cucurbituril Homologues and Derivatives: New Opportunities in Supramolecular Chemistry. Acc. Chem. Res..

[32] Kim K. (2019). Cucurbiturils and Related Macrocycles.

[33] El-Sheshtawy H.S., Chatterjee S., Assaf K.I., Shinde M.N., Nau W.M., Mohanty J. (2018). A Supramolecular Approach for Enhanced Antibacterial Activity and Extended Shelf-Life of Fluoroquinolone Drugs with Cucurbit[7]Uril. Sci. Rep..

[34] Lü J., Lin J.X., Cao M.N., Cao R. (2013). Cucurbituril: A Promising Organic Building Block for the Design of Coordination Compounds and Beyond. Coord. Chem. Rev..

[35] Assaf K.I., Nau W.M. (2015). Cucurbiturils: From Synthesis to High-Affinity Binding and Catalysis. Chem. Soc. Rev..

[36] Nau W.M. (2010). Supramolecular Capsules: Under Control. Nat. Chem..

[37] Jeon W.S., Moon K., Park S.H., Chun H., Ko Y.H., Lee J.Y., Lee E.S., Samal S., Selvapalam N., Rekharsky M.V. (2005). Complexation of Ferrocene Derivatives by the Cucurbit[7]Uril Host: A Comparative Study of the Cucurbituril and Cyclodextrin Host Families. J. Am. Chem. Soc..

[38] Shaikh M., Mohanty J., Singh P.K., Nau W.M., Pal H. (2008). Complexation of Acridine Orange by Cucurbit[7]Uril and β-Cyclodextrin: Photophysical Effects and PKa Shifts. Photochem. Photobiol. Sci..

[39] Singh M.K., Pal H., Koti A.S.R., Sapre A.V. (2004). Photophysical Properties and Rotational Relaxation Dynamics of Neutral Red Bound to β-Cyclodextrin. J. Phys. Chem. A.

[40] Zonjić I., Radić Stojković M., Crnolatac I., Tomašić Paić A., Pšeničnik S., Vasilev A., Kandinska M., Mondeshki M., Baluschev S., Landfester K. (2022). Styryl Dyes with *N*-Methylpiperazine and *N*-Phenylpiperazine Functionality: AT-DNA and G-Quadruplex Binding Ligands and Theranostic Agents. Bioorg. Chem..

[41] Chernikova E.Y., Ruleva A.Y., Tsvetkov V.B., Fedorov Y.V., Novikov V.V., Aliyeu T.M., Pavlov A.A., Shepel N.E., Fedorova O.A. (2020). Cucurbit[7]Uril-Driven Modulation of Ligand-DNA Interactions by Ternary Assembly. Org. Biomol. Chem..

[42] Tian T., Song Y., Wei L., Wang J., Fu B., He Z., Yang X.R., Wu F., Xu G., Liu S.M. (2017). Reversible Manipulation of the G-Quadruplex Structures and Enzymatic Reactions through Supramolecular Host-Guest Interactions. Nucleic Acids Res..

[43] Perevozchikova P.S., Chernikova E.Y., Shepel N.E., Fedorova O.A., Fedorov Y.V. (2023). DNA-Based Assemblies with Bischromophoric Styryl Dye-Chromene Conjugates and Cucurbit[7]Uril. Spectrochim. Acta Part A Mol. Biomol. Spectrosc..

[44] Gavvala K., Satpathi S. (2016). Acetylcholine Induced Interplay of Proflavine between Cucurbit[7]Uril and DNA. J. Lumin..

[45] Kircheva N., Dobrev S., Dasheva L., Koleva I., Nikolova V., Angelova S., Dudev T. (2020). Complexation of Biologically Essential (Mono- and Divalent) Metal Cations to Cucurbiturils: A DFT/SMD Evaluation of the Key Factors Governing the Host–Guest Recognition. RSC Adv..

[46] Koleva I.Z., Dobrev S., Kircheva N., Dasheva L., Nikolova V., Angelova S., Dudev T. (2022). Complexation of Trivalent Metal Cations (Al3+, Ga3+, In3+, La3+, Lu3+) to Cucurbiturils: A DFT/SMD Evaluation of the Key Factors Governing the Host-Guest Recognition†. Phys. Chem. Chem. Phys..

[47] Kircheva N., Dobrev S., Dasheva L., Nikolova V., Angelova S., Dudev T. (2023). Metal-Assisted Complexation of Fluorogenic Dyes by Cucurbit[7]Uril and Cucurbit[8]Uril: A DFT Evaluation of the Key Factors Governing the Host–Guest Recognition. Molecules.

[48] Kircheva N., Nikolova V., Dobrev S., Angelova S., Dudev T. (2022). β -Cyclodextrin-Modulated Interaction of Gd^3 +^ with Levofloxacin: A Molecular Modeling Study. Trends Phys. Chem..

[49] Nikolova V., Dobrev S., Kircheva N., Yordanova V., Dudev T., Angelova S. (2023). Host-Guest Complexation of Cucurbit[7]Uril and Cucurbit[8]Uril with the Antimuscarinic Drugs Tropicamide and Atropine. J. Mol. Graph. Model..

[50] Mohanty J., Thakur N., Dutta Choudhury S., Barooah N., Pal H., Bhasikuttan A.C. (2012). Recognition-Mediated Light-up of Thiazole Orange with Cucurbit[8]Uril: Exchange and Release by Chemical Stimuli. J. Phys. Chem. B.

[51] Choudhury S.D., Mohanty J., Pal H., Bhasikuttan A.C. (2010). Cooperative Metal Ion Binding to a Cucurbit[7]Uril—Thioflavin T Complex: Demonstration of a Stimulus-Responsive Fluorescent Supramolecular Capsule. J. Am. Chem. Soc..

[52] Shaikh M., Choudhury S.D., Mohanty J., Bhasikuttan A.C., Pal H. (2010). Contrasting Guest Binding Interaction of Cucurbit[7-8]Urils with Neutral Red Dye: Controlled Exchange of Multiple Guests. Phys. Chem. Chem. Phys..

[53] Biedermann F., Uzunova V.D., Scherman O.A., Nau W.M., De Simone A. (2012). Release of High-Energy Water as an Essential Driving Force for the High-Affinity Binding of Cucurbit[n]Urils. J. Am. Chem. Soc..

[54] Jeon Y.M., Kim J., Whang D., Kim K. (1996). Molecular Container Assembly Capable of Conrolling Binding and Release of Its Guest Molecules: Reversible Encapsulation of Organic Molecules in Sodium Ion Complexed Cucurbituril. J. Am. Chem. Soc..

[55] Shen F.F., Zhao J.L., Chen K., Hua Z.Y., Chen M.D., Zhang Y.Q., Zhu Q.J., Tao Z. (2017). Supramolecular Coordination Assemblies of a Symmetrical Octamethyl-Substituted Cucurbituril with Alkali Metal Ions Based on the Outer-Surface Interactions of Cucurbit[: N] Urils. CrystEngComm.

[56] Yao Y.Q., Chen K., Hua Z.Y., Zhu Q.J., Xue S.F., Tao Z. (2017). Cucurbit[n]Uril-Based Host–Guest-Metal Ion Chemistry: An Emerging Branch in Cucurbit[n]Uril Chemistry. J. Incl. Phenom. Macrocycl. Chem..

[57] Zhang S., Grimm L., Miskolczy Z., Biczók L., Biedermann F., Nau W.M. (2019). Binding Affinities of Cucurbit[: N] Urils with Cations. Chem. Commun..

[58] Ko Y.H., Kim K., Kang J.K., Chun H., Lee J.W., Sakamoto S., Yamaguchi K., Fettinger J.C., Kim K. (2004). Designed Self-Assembly of Molecular Necklaces Using Host-Stabilized Charge-Transfer Interactions. J. Am. Chem. Soc..

[59] Ni X.L., Lin J.X., Zheng Y.Y., Wu W.S., Zhang Y.Q., Xue S.F., Zhu Q.J., Tao Z., Day A.I. (2008). Supramolecular Bracelets and Interlocking Rings Elaborated through the Interrelationship of Neighboring Chemical Environments of Alkyl-Substitution on Cucurbit[5]Uril. Cryst. Growth Des..

[60] Zhang F., Yajima T., Li Y.Z., Xu G.Z., Chen H.L., Liu Q.T., Yamauchi O. (2005). Iodine-Assisted Assembly of Helical Coordination Polymers of Cucurbituril and Asymmetric Copper(II) Complexes. Angew. Chem. Int. Ed..

[61] Chandra F., Dutta T., Koner A.L. (2020). Supramolecular Encapsulation of a Neurotransmitter Serotonin by Cucurbit[7]Uril. Front. Chem..

[62] Kircheva N., Dobrev S., Petkova V., Bakalova S., Kaneti J., Angelova S. (2023). Theoretical Assessment of the Ligand/Metal/Quadruplex Recognition in the Non-Canonical Nucleic Acids Structures. Molecules.

[63] Frisch M., Trucks G.W., Schlegel H.B., Scuseria G.E., Robb M.A., Cheeseman J.R., Scalmani G., Barone V., Mennucci B., Petersson G.A. (2013). Gaussian 09, Revision d. 01.

[64] Zhao Y., Truhlar D.G. (2008). The M06 Suite of Density Functionals for Main Group Thermochemistry, Thermochemical Kinetics, Noncovalent Interactions, Excited States, and Transition Elements: Two New Functionals and Systematic Testing of Four M06-Class Functionals and 12 Other Function. Theor. Chem. Acc..

[65] Nikolova V., Velinova A., Dobrev S., Kircheva N., Angelova S., Dudev T. (2021). Host–Guest Complexation of Cucurbit[7]Uril and Cucurbit[8]Uril with the Antineoplastic and Multiple Sclerosis Agent Mitoxantrone (Novantrone). J. Phys. Chem. A.

[66] Cao L., Škalamera Đ., Zavalij P.Y., Hostaš J., Hobza P., Mlinarić-Majerski K., Glaser R., Isaacs L. (2015). Influence of Hydrophobic Residues on the Binding of CB[7] toward Diammonium Ions of Common Ammonium⋯ammonium Distance. Org. Biomol. Chem..

[67] Bardelang D., Udachin K.A., Leek D.M., Margeson J.C., Chan G., Ratcliffe C.I., Ripmeester J.A. (2011). Cucurbit[n]Urils (n = 5–8): A Comprehensive Solid State Study. Cryst. Growth Des..

[68] McQuire D.A., Simon J.D. (1997). Physical Chemistry: A Molecular Approach.

[69] Marenich A.V., Cramer C.J., Truhlar D.G. (2009). Universal Solvation Model Based on Solute Electron Density and on a Continuum Model of the Solvent Defined by the Bulk Dielectric Constant and Atomic Surface Tensions. J. Phys. Chem. B.

[70] Schrödinger L., DeLano W. (2020). PyMOL. http://www.pymol.org/pymol.

