# Supplementary material for: N-Methyl- and N-Phenylpiperazine Functionalized Styryl Dyes Inside Cucurbiturils: Theoretical Assessment of the Factors Governing the Host–Guest Recognition
Source: Molecules. 2023 Dec 16;28(24):8130. doi: 10.3390/molecules28248130 (PMC10746092; doi:10.3390/molecules28248130)
Supplement: Supplementary file 1 [file molecules-28-08130-s001.zip › molecules-2754732-supplementary.pdf]

molecules

Supplementary Materials

N-Methyl- and *N*-Phenylpiperazine Functionalized Styryl Dyes inside Cucurbiturils: Theoretical Assessment of the Factors Governing the Host-Guest Recognition

Nikoleta Kircheva [1], Vladislava Petkova [1], Stefan Dobrev [1], Valya Nikolova [2], Silvia Angelova [1,3], Todor Dudev [2,*]

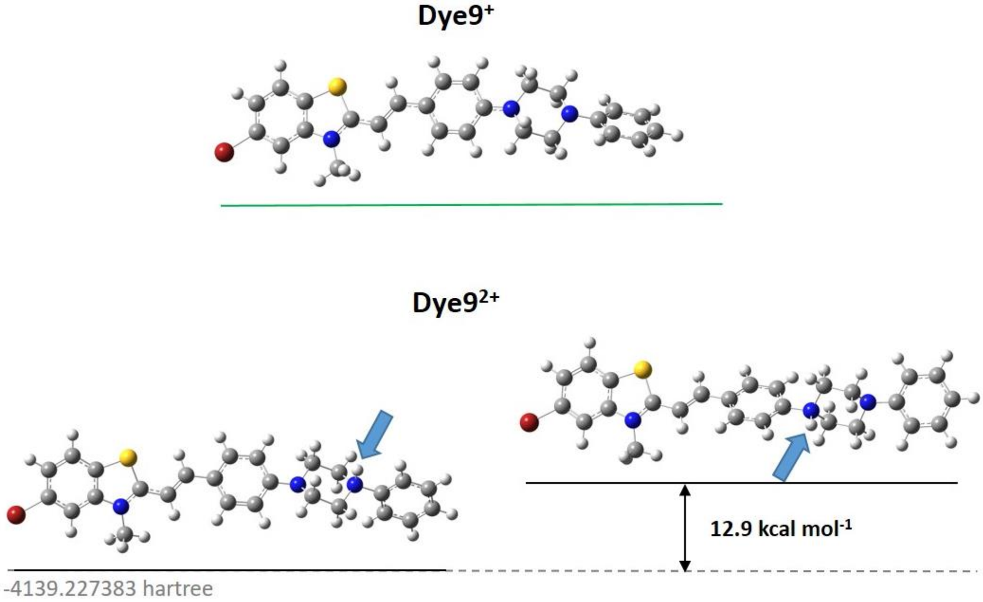

Figure S1. Optimized structures of dye9 in its mono- (Dye9^{+}) and dicationic (Dye9^{2+}) forms. The energetically preferred form of Dye9^{2+} is the N1-protonated (left).
